# CREB: A Key Regulator of Normal and Neoplastic Hematopoiesis

**DOI:** 10.1155/2009/634292

**Published:** 2009-08-27

**Authors:** Salemiz Sandoval, Martina Pigazzi, Kathleen M. Sakamoto

**Affiliations:** ^1^Division of Hematology/Oncology, Department of Pediatrics, Gwynne Hazen Cherry Memorial Laboratories, Mattel Children's Hospital, Jonsson Comprehensive Cancer Center, Department of Pathology and Laboratory Medicine, David Geffen School of Medicine, Molecular Biology Institute, CA Nanosystems Institute, UCLA, Los Angeles, CA 90095, USA; ^2^Department of Pediatrics, Laboratory of Pediatric Onco-hematology, University of Padova, 35128 Padova, Italy

## Abstract

The cAMP response element-binding protein (CREB) is a nuclear transcription factor downstream of cell surface receptors and mitogens that is critical for normal and neoplastic hematopoiesis. Previous work from our laboratory demonstrated that a majority of patients with acute myeloid leukemia (AML) and acute lymphoid leukemia (ALL) overexpress CREB in the bone marrow. To understand the role of CREB in leukemogenesis, we examined the biological effect of CREB overexpression on primary leukemia cells, leukemia cell lines, and CREB overexpressing transgenic mice. Our results demonstrated that CREB overexpression leads to an increase in cellular proliferation and survival. Furthermore, CREB transgenic mice develop a myeloproliferative disorder with aberrant myelopoiesis in both the bone marrow and spleen. Additional research from other groups has shown that the expression of the cAMP early inducible repressor (ICER), a CREB repressor, is also deregulated in leukemias. And, miR-34b, a microRNA that negative regulates CREB expression, is expressed at lower levels in myeloid leukemia cell lines compared to that of healthy bone marrow. Taken together, these data suggest that CREB plays a role in cellular transformation. The data also suggest that CREB-specific signaling pathways could possibly serve as potential targets for therapeutic intervention.

## 1. Introduction

CREB is a 43-kDa protein, memberof the CREB/ATF-1 family of transcription factors, conserved from *Drosophila* to humans [1]. In mammals, CREB family members include CREB, cAMP-responsive element modulator (CREM), and activating transcription factor 1 (ATF-1). CREB and ATF-1 are ubiquitously expressed in all tissues. The expression of CREM, however, is tissue specific and developmentally regulated. This family of transcription factors contains a 60 amino acid kinase-inducible domain (KID) with several phosphorylation sites, two hydrophobic glutamine-rich transactivation domains, Q1 and Q2, that function as constitutive activators in vitro, and a basic leucine zipper (bZip) dimerization domain. CREB is activated through phosphorylation at serine 133 in response to a variety of cellular and mitogen stress signals. These include peptide hormones, neurotransmitters, calcium influx, and growth factors [1–3]. Upon activation, CREB binds as a dimer to the cAMP response element (CRE), TGACGTCA, or CRE half sites CGTCA/TGACG, where it promotes the recruitment of the transcriptional coactivators CREB binding protein (CBP) and p300. These coactivators then promote the recruitment of components of the basal transcriptional machinery to initiate transcription of CREB target genes [3–5]. 

 CREB activity is also regulated by a family of cytoplasmic coactivators known as transducers of regulated CREB activity (TORCs). There are three TORC members, TORC1, TORC2, and TORC3, and all are thought to be strong activators of CREB-dependent transcription. These coactivators bind the bZip domain of CREB in response to extracellular stimuli, such as cAMP, calcium, and hormones. When activated, TORCs are translocated into the nucleus where they activate CREB through a phosphoserine-133 independent mechanism [6, 7]. 

 CREB has been implicated in a number of cellular events. CREB coactivators help discriminate between signals to activate only specific cellular processes. However, it has been demonstrated that the interaction between CREB and its coactivators is far too weak to activate transcription of CREB target genes [1]. This suggests that many CREB interacting proteins are yet to be identified. Thus, future research should focus on identifying CREB interacting partners that further potentiate CREB activation or confer signal specificity. 

## 2. CREB Kinases

**Figure 1:**
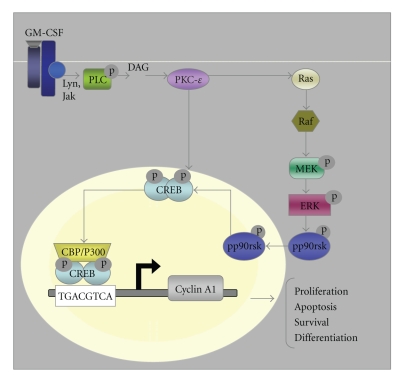
*Pathways for CREB activation in hematopoietic cells*. A variety of extracellular stimuli promote CREB activation through phosphorylation or through interaction with CREB coactivators to enhance the expression of CREB responsive genes. CREB target genes have been shown to mediate effects on cellular proliferation, apoptosis, survival, and differentiation. PLC : phospholipase-C, DAG : 1,2-diacylglycerol, PKC-*ε* : protein kinase C-*ε*.

There are several serine/threonine kinases that have been reported to activate CREB. Stimuli such as cAMP, calcium, and growth factors and cellular stress activate kinases such as ribosomal protein S6 kinase (pp90rsk), protein kinase A (PKA), protein kinase C (PKC), protein kinase B/AKT, and (mitogen- and stress-activated protein kinase) MSK-1, subsequently activate CREB [2]. Mitogenic and ultraviolet stress, for example, lead to the activation of mitogen/stresses activated kinase-1 (MSK-1), a pp90rsk family member that in turn phosphorylates CREB [8]. CREB phosphorylation is abrogated in fibroblasts from MSK-1/MSK-2 double knockout mice when stimulated with mitogens or when it is under cellular stress [9]. CREB is also phosphorylated in response to Granulocyte-Macrophage Colony Simulating Factor (GM-CSF) by pp90rsk in myeloid cells (Figure 1), leading to the activation of immediate early genes, c-fos, activator protein 1 (AP-1/junB), and early growth response protein 1 (egr-1) [10]. And CREB activation by MAPK and AKT/B enhances the survival of cultured cells [11]. In 2002, Raes and others characterized a new signaling pathway leading to CREB activation. Using L929 murine fibrosarcoma and *ρ*0 143B human osteosarcoma cell lines, this group demonstrated that CREB was phosphorylated by Calmodulin kinase IV (CAMKIV) as a consequence of mitochondrial dysfunction. In these mitochondrially impaired cells, CREB is constitutively activated as a result of high intracellular calcium levels that disrupt the association between CaMKIV and the protein phosphatase 2A (PP2A), resulting in constitutively activated CAMKIV [12]. These CREB kinases are among a long list of kinases that activate CREB in response of many extracellular stimuli. 

## 3. CREB Activates Cellular Targets with Diverse Sets of Functions

CREB has been implicated in a great number of cellular functions, including metabolism, proliferation, apoptosis, and differentiation. Studies have demonstrated that CREB is phosphorylated in response to up to 300 different stimuli [3]. And upon activation, CREB enhances the expression of up to 5000 putative genes [5]. More stringent genome wide analysis for CREB binding motifs identified 1349 mouse and 1663 human CREB binding sites [13]. A quarter of the CRE-containing sequences function in cellular metabolism. In the liver, for example, CREB regulates gluconeogenesis, through phosphoenol pyruvate carboxykinase [14, 15]. Other metabolic enzymes, such as pyruvate carboxylase, ornithine decarboxylase, and lactate dehydrogenase contain CRE consensus sites in their promoters [2]. It has also been well characterized that CREB plays a critical role in survival. In sympathetic and cerebral neurons, nerve growth factor (NGF) and brain-derived neurotrophic factor (BDNF) stimulate survival by activating the expression of the antiapoptotic protein B-cell lymphoma 2 (bcl-2) [16, 17]. This was further shown by overexpression of a dominant negative form of CREB in these cells resulting in increased cell death. Moreover, this effect was reverted by overexpression of bcl-2. CREB has also been shown to regulate proliferation through cyclins A1 and D1 [2, 18, 19]. For instance, knockdown of CREB in both TF-1 and K562 cells leads to a decrease in the expression of cell cycle regulators, Cyclin A1 (Figure 1) and Cyclin D1. 

 Furthermore, CRE-binding proteins have been shown to play a role in the physiology of the pituitary gland, in regulating spermatogenesis and circadian rhythm, and in learning and memory [20]. The functional diversity of the proteins CREB regulates suggests that cell surface proteins or CREB signaling molecules must confer specificity or allow for discrimination between signal inputs to activate particular cellular processes. 

## 4. Role of CREB in Cancer

Many of the CREB target genes identified function in cell growth, survival, and cell-cycle regulation, and their aberrant expression has been associated with cancer. The genes identified include genes that enhance proliferation, cell cycle progression, and survival, such as c-fos, cyclins A1 and D1, and survival, such as the bcl-2 protein. A role for CREB family members in cancer was first identified in clear-cell sarcomas of the soft tissues (CSSTs). Most CSSTs contain the chromosomal translocation t(12;22)(q13;q12) that fuses the DNA-binding and bZip domain of ATF1 to the Ewing's sarcoma gene, EWS. The EWS-ATF1 fusion protein promotes proliferation and inhibits apoptosis [21–23]. Recently, an EWS-CREB fusion protein was also identified in a subset of CSST patients [24]. EWS-ATF1 and EWS-CREB fusion onco-proteins are constitutively active and enhance the expression of CREB target genes, independent of growth or signal stimulus. 

 Interestingly, CREB is also required for viral transformation of T lymphocytes by the oncogenic retrovirus human T-cell leukemia virus (HTLV-I). HTLV-I causes adult T cell leukemia, an aggressive neoplasm that results in the increase in the responsiveness of cells to extracellular stimuli enhancing their proliferation and survival while inhibiting apoptosis. Tax, an oncogenic viral protein of HTLV-I, physically interacts with CREB to potentiate CREB dimerization and DNA binding to allow for the recruitment of the coactivators p300 and CBP in the absence of serine-133 phosphorylation [25]. Tax has also been shown to bind TORCs to potentiate CREB activation and increase the transcription of viral and cellular targets of CREB [26, 27]. Another oncogenic retrovirus, Hepatitis B virus (HBV), has also been shown to promote cellular transformation by enhancing CREB target gene expression in a similar way to HTLV-1. HBV interacts with CREB/ATF2 and p300/CBP to constitutively turn on CREB genes [28]. 

 TORCs, CREB coactivators, have also been implicated in cancers. TORCs are deregulated in mucoepidermoid carcinomas, a salivary gland tumor that possesses a t(11;19)(q14–21;p12–13) translocation. This translocation fuses the N-terminal amino acids of TORC1 to the epidermoid carcinoma translocated-1 (METC-1) to the transcriptional activation domain of the Notch co-activator Mastermind-like 2 (MAML2). The TORC1-MAML2 fusion protein retains the CREB binding domain, which activates the transcription of CREB cellular targets [6, 29]. It has been well documented that TORC1-MAML2 but not TORC1 or MAML2 can transform cells [30]. Moreover, a dominant form of CREB, lacking the ability to bind DNA, cannot transform cells [31]. 

 CREB has also been implicated in many other cancers, some of which include hepatocellular carcinoma, osteosarcoma, lung adenocarcinoma, and leukemias [32]. This evidence suggests that CREB as a fusion protein or by cooperation with other oncogenes can promote cellular transformation [33].

## 5. CREB is Key Regulator of Hematopoiesis and Leukemogenesis

Hematopoiesis involves the expansion and differentiation of a small population of pluripotent stem cells into progenitor cells. Maturation of these stem cells requires the exposure of these cells to a combination of differentiation and growth signals. CREB is downstream of cell surface receptors and mitogens that control normal hematopoiesis and leukemogenesis. Recent studies have demonstrated that CREB modulates the expression of genes that regulate hematopoiesis [20, 34]. In the human erythroid cell line, TF-1 for example, CREB was shown to regulate the expression of immediate early gene, egr-1 [10, 35]. In these cells, interleukin-3 (IL-3) and GM-CSF signaling results in CREB activation of egr-1 in response to pp90RSK activation through a Mitogen-activated protein/extracellular regulated kinase- (MEK-) dependant signaling pathway. Other studies have shown that IL-3 and GM-CSF, but not IL-4, potentiate CREB activation through PKC-*ε* in TF-1 cells [36]. Egr-1 is critical for transcription of myeloid-specific proteins that function as determinants of myeloid cell proliferation and differentiation. CREB also appears to play a role in megakaryocytic differentiation [36]. Studies performed in the biphenotypic cell line, HEL (erythroid/megakaryocytic), and CD34^+^ cells from normal patients show that thrombopoietin (TPO), and forskolin (FK), and phorbol myristate acetate (PMA) leads to increased activation of CREB through a Mitogen-activated protein kinase- (MAPK-) dependent mechanism [37]. 

 Our group has provided additional evidence for the role of CREB in hematopoiesis. Expression analysis of both murine and human primary cells revealed that CREB is highly expressed in stem cells and uncommitted progenitors [19]. A 2.6-fold increase in CREB expression was observed in committed marker lineage-negative stem cells and progenitors compared to differentiated lineage-positive murine bone marrow cells. Further fractionation of bone marrow cells showed that CREB expression was increased in hematopoietic stem cells (HSCs), common myeloid progenitors (CMPs), granulocyte-macrophage progenitors (GMPs), and megakaryocytic erythroid progenitors (MEPs). Analysis of human stem cells confirmed these results, showing that CREB expression was increased by twofold in immature committed marker lineage-negative, CD34 positive hematopoietic cells compared to differentiated committed marker lineage-positive CD34 negative hematopoietic cells from both cord and peripheral blood [19]. 

 To further investigate the requirement of CREB in normal hematopoiesis, normal murine bone marrow and human peripheral blood stem cells (PBSCs) were transduced with CREB lentiviral shRNAs. Colony assays demonstrated that CREB inhibition affected growth and increased apoptosis in knockdown cells compared to vector controls [19]. CREB downregulation also significantly decreased the number of CFU-GM colonies in both murine bone marrow cells and human PBSCs. Collectively, these data demonstrate that CREB downregulation results in abnormal proliferation, survival, and cell-cycle regulation of normal hematopoietic cells. 

 In addition to playing a role in normal hematopoiesis, CREB has also been shown to potentiate transformation of hematopoietic cells. As mentioned earlier, CREB has been linked to the pathogenesis of HTLV-1 leukemias by Tax, a viral transcription factor that enhances CREB activation [32, 33]. CREB has also been implicated in the pathogenesis of lymphomas. CREB activates the transcription of bcl-2, an antiapoptotic protein in follicular lymphomas bearing the t(14;18) translocation [38, 39]. Studies show that CREB binds and enhances the expression of bcl-2 through altered allele but not the normal allele in follicular and transformed lymphomas. 

 Additionally, the CREB coactivators, CBP and p300, have also been associated hematologic malignancies [40, 41]. For example, CBP is fused to the transcription factor (monocytic leukemia zinc finger) MOZ in M4 and M5 leukemias bearing the translocation t(8;16)(p11;13) [42]. And the chromosomal translocation t(11;16), which is associated with acute and chronic myeloid leukemias, fuses (Myeloid/lymphoid or mixed-lineage leukemia) the MLL transcriptional regulator to CBP [43–46]. Together, these findings highlight the importance of CREB and CREB regulatory factors in modulating growth, survival, and transformation of hematopoietic cells. 

 Leukemia is one of the most common forms of cancer. This hematologic malignancy is characterized by the acquisition of recurring mutations and gene rearrangements that lead to aberrant proliferation or survival advantage in undifferentiated cells. To study the possible role of CREB in leukemogenesis, we analyzed primary bone marrow cells for CREB expression in patients with acute leukemia. Our results demonstrated that a majority of patients with acute myeloid leukemia and acute lymphoid leukemia overexpress CREB two- to threefold in the bone marrow at diagnosis and relapse but not in remission or nonleukemic controls [18, 47]. Since CREB was upregulated at both the protein and mRNA level in CREB-positive (CREB+) primary AML cells, we hypothesized that CREB could be amplified at the genomic level. Fluorescent in situ hybridization (FISH) was performed on blast cells from four CREB-overexpressing patients using a CREB-specific bacterial artificial chromosome clone. In the blast cells from three out of four CREB+ AML patients, we detected three to four signals from one homolog and one signal from the other homolog in over 250 interphase nuclei analyzed [18]. These results indicate that certain patients have more than the two normal copies of CREB, which may be one potential mechanism for CREB overexpression. However, the nature of this amplification has not yet been determined. AML patients that overexpress CREB are associated with an increased risk of relapse and a decreased in event-free survival compared to patients that do not overexpress CREB. Studies performed in leukemia cell lines demonstrated that CREB overexpression results in increased proliferation and increased survival of these cells in the absence of growth factors. Elevated CREB levels also resulted in decreased differentiation of K562 cells treated with sodium butyrate, while downregulation of endogenous CREB by siRNA decreased survival and proliferation of leukemia cell lines, suggesting that CREB modulates growth and survival of myeloid leukemia cells. Moreover, mice overexpressing CREB in macrophage/monocyte lineage cells develop a myeloproliferative disease/myelodysplastic syndrome with higher white blood counts and aberrant myelopoiesis in both the bone marrow and spleen after one year. However, these mice did not develop AML. Additionally, bone marrow progenitor cells from CREB transgenic mice exhibit properties of transformed cells, such as increased proliferation, immortalization, and hypersensitivity to growth factors in colony assays. 

 We also examined the requirement of CREB in malignant hematopoiesis by lentiviral shRNA downregulation of CREB in several leukemia cell lines. Inhibition of CREB in these cell lines suppresses leukemic cell proliferation in vitro [19]. In vivo results, correspondingly, show that downregulation of CREB in the Bcr-abl positive BA/F3 leukemia cells injected into Severe Combined Immunodeficient (SCID) mice inhibited early leukemic progression or cell proliferation in mice, resulting in a prolonged median survival of these mice compared to control mice. These data suggest that CREB is critical for progression of disease. However, these results also demonstrate that CREB contributes to but is not sufficient for leukemogenesis, and that additional mutations are necessary for the development of leukemia. 

 Other groups have confirmed the role of CREB in leukemogenesis. Pigazzi et al. investigated CREB expression in acute leukemia patients and found that 84% (73/86) patients with ALL and 80% (32/40) patients with AML overexpressed CREB by Western blot and ELISA analysis [48]. They also observed that CREB was phosphorylated and thus active at diagnosis but not in remission or in non-leukemic controls. Furthermore, using gel shift assays, they demonstrated that CREB binds the CRE consensus site in bone marrow cells from leukemia patients at diagnosis, but not in remission or control samples. 

 The Pigazzi group also examined the expression of the cAMP early inducible repressor (ICER). This transcriptional regulator represses CREB activity by competing for the CRE consensus site. Analysis of ICER expression showed that ICER was downregulated at diagnosis but was significantly increased in remission and control samples [49]. This group further investigated ICER and its role in inhibiting leukemic transformation by expressing ICER in HL60 and HeLa cancer cell lines and examining colony formation *in vitro*. ICER transfectants were found to have a significantly lower amount of colonies compared to control cells. Chromatin immunoprecipitation experiments showed that ICER recognizes and competes for CRE elements opposing CREB target gene expression of Signal Transducers and Activator of Transcription-3 (STAT-3), p21, fos and bcl-2. 

 Additionally, in vivo experiments showed that ICER expression in HL60 cells affects cellular transformation [49]. Mice were inoculated with HL60 cells and HL60-ICER transfectants and bone marrow engraftment and invasion of extramedullary sites was examined. Results showed that engraftment was unaffected, however, invasion into the peripheral blood and spleen was greatly reduced in HL60-ICER mice compared to controls. Moreover, they showed that HL60-ICER cells had decreased angiogenic potential in vivo as they exhibited a decrease in the amount of microvessel numbers and size. 

## 6. Mir-34b Regulation of CREB

The mechanism of CREB overexpression in leukemia remains unknown. MicroRNAs (miRNAs) are small noncoding RNAs that can either positively or negatively regulate gene expression principally through translational repression and targeting mRNA for degradation. There is recent evidence that microRNAs also regulate CREB expression. Approximately 30% of human genes possess conserved miRNA binding sites and are presumed to be regulated by microRNAs [50]. Generally, miRNAs bind mRNA sequences located at the 3’-untranslated region (UTR) with imperfect complementarity. MicroRNAs avoid target mRNA on polysomes, that would lead to a block in translation or mRNA degradation. The mRNA partners of microRNAs, microRNA function, and their tissue specificity are currently being investigated in normal and diseased patient samples to increase our understanding of tumorigenesis. 

 MicroRNAs have been demonstrated to control a variety of cellular pathways by influencing the expression of specific target genes [51, 52]. Hundreds of miRNAs have been identified to date, but their specific functions and target mRNAs have been assigned for only a few [53]. MiRNA expression has been demonstrated to be tissue specific, and to control cellular differentiation, proliferation and survival, and changes in their expression have been associated with many diseases, including human cancer [54]. A role for miRNAs in several tumors has recently been recognized, with intrinsic tumor suppressor or oncogenic functions. There is considerable evidence that suggest a crucial role for miRNAs in chronic lymphocytic leukemia [55, 56], and an involvement of miR-223 and miR-155 has already been proposed in the pathogenesis of AML [57, 58]. Inappropriate expression of candidate miRNAs that potentially bind to the CREB gene has been evaluated by Pigazzi group [59]. They found that miR-34b a possible candidate to target CREB by in silico and gene expression analyses. MiR-34b was expressed at lower levels in myeloid leukemia cell lines compared to healthy bone marrow, in agreement with other experiments that described low levels of the expression of the miR-34 family of miRNA in other human cancers [60]. The restoration of miR-34b revealed a direct interaction with the CREB-3’UTR, with reduction of the CREB protein levels in vitro. MiR-34b was demonstrated to induce cell cycle abnormalities, reduced anchorage independent growth, and altered CREB target gene expression, suggesting its potential to act as a tumor suppressor. The molecular mechanism by which miR-34 family of miRNAs suppress tumors is currently under investigation for many cancers [61, 62]. Until now, gene expression analyses have been performed, suggesting that the cause of tumor suppression might be the ability to target genes related to the cell cycle pathway. Pigazzi et al. described many of the CREB target proteins, for example, BCL-2, Cyclins A1, B1, D, NfKB, JAK1, STAT3, as well as many downstream protein kinases, and cell survival signaling pathways, for example, AKT/mTOR, ERK, are decreased. 

 At this time, there is no evidence of miR-34b downregulation, apart from the frequent deficiency of functional p53 that drives their transcription in several cancer cells. Colorectal cancer studies previously identified a methylated miR-34b/34c promoter at chromosome 11q23, which might have reduced p53 transcriptional activity due to deletion of this region [63]. Treatment of AML cell lines with a demethylating agent and studies of the CpG island at the miR-34b/34c promoter confirmed that methylation might be one of the mechanisms of miR-34b downregulation. Moreover, demethylation treatment leads to a decrease in CREB protein levels, suggesting that miR-34b is epigenetically modified in myeloid leukemia cells to maintain tumor progression [63]. The clinical relevance of the in vitro experiments was substantiated by data obtained in AML patients. In fact, expression levels of miR-34b were decreased in AML patients compared to healthy bone marrow samples. Decreased expression of miR-34b also correlated with increased levels of CREB protein confirming the relationship between CREB expression and miRNA regulation.

## 7. Concluding Remarks

In summary, we conclude that CREB is a proto-oncogene whose overexpression can potentiate transformation of hematopoietic cells. However, CREB's biologic effect on proliferation and survival is not sufficient to induce leukemias in vivo. Similar observations have been described with other transgenic mouse models of leukemia. AML1-ETO, K-Ras and FLT3-internal tandem duplication (ITD) transgenic mice, for example, develop myeloproliferative disorders but not leukemias. Similarly, CREB in vivo contributes to the leukemia phenotype, but additional mutations or ‘hits’ are required for disease development. 

 This review discusses the role of CREB in cellular transformation. How CREB target gene activation leads to the transforming phenotype, however, is yet to be fully understood. CREB has been shown to associate with a number of cellular coactivators. However, there is evidence that the interactions between CREB and its coactivators are too weak to activate transcription of CREB target genes. This suggests that many CREB interacting proteins are yet to be identified. Future research will focus on identifying additional CREB interacting proteins and the target genes that confer a proliferative or survival advantage to myeloid cells resulting in transformation. Once better understood, CREB-specific signaling molecules could serve as potential targets for therapeutic intervention.
